# Agency, time, and causality

**DOI:** 10.3389/fpsyg.2014.01264

**Published:** 2014-11-06

**Authors:** Thomas Widlok

**Affiliations:** Kulturanthropologie Afrikas, Institut für Afrikanistik, Global South Studies Center, Universität zu KölnKöln, Germany

**Keywords:** causality, time, agency, ethnography, Africa

## Abstract

Cognitive Scientists interested in causal cognition increasingly search for evidence from non-Western Educational Industrial Rich Democratic people but find only very few cross-cultural studies that specifically target causal cognition. This article suggests how information about causality can be retrieved from ethnographic monographs, specifically from ethnographies that discuss agency and concepts of time. Many apparent cultural differences with regard to causal cognition dissolve when cultural extensions of agency and personhood to non-humans are taken into account. At the same time considerable variability remains when we include notions of time, linearity and sequence. The article focuses on ethnographic case studies from Africa but provides a more general perspective on the role of ethnography in research on the diversity and universality of causal cognition.

## INTRODUCTION

Scientific enquiry during much of the 19th and 20th century searched for “weird” examples of human cognition in non-European societies and cultures, typically highlighting extreme departures from Western societies that were considered to be the “standard” (see for example Porteus, 1937). A programmatic turning point in this orientation has recently been marked by Henrich et al. (2010) who noted that much of experimental psychology (and economics) to date is limited by a sample that is made up of “weird” outliers of a different sort, namely a sample of university students conscripted to experiments and of similar subjects from a Western Educational Industrial Rich Democratic (WEIRD) background. It has become clear that a good number of foundational experiments exhibit such a bias since the WEIRD subjects’ responses are very different from those of other populations. As soon as subjects with a broader cultural background are included, some presumed cognitive universals (such as the Müller-Lyer illusion) turn out to occur only in some populations (Henrich et al., 2010, p. 65). As a consequence the preparedness to include “non-WEIRD” groups has grown and is increasingly considered to be obligatory, except that there is often only a very vague sense as to what exactly such a desirable broadening of the sample should look like. Is it enough to include non-Europeans who were initially only considered when extreme contrasts were sought after? For instance, members of the Sudanese Zande, Nuer, and Dinka, people that feature in this contribution, are likely to be considered prototypical examples of a non-WEIRD population because they live in Africa and they have so far featured in ethnographic writing rather than in cognitive experimenting.

However, the matter is less straightforward than may initially appear to be the case. Although originally African people, there is a considerable “Western” diaspora of Nuer living in the USA with considerable economic and cultural effects on the Nuer remaining in Africa (Falge, 2006), Dinka migrants are numerous enough to make them a recognized immigrant category in Australia^1^ and they are frequent participants in diaspora blogs^2^. Furthermore, Nuer and Dinka organizations have been in the media spotlight of recent conflicts and have themselves created an internet presence^3^, suggesting that at least a good proportion of the members of these groups are also “educated,” at least computer literate. Although Southern Sudan is currently best known for its food crisis and shortage of products, one of the main causes for this situation is the conflict about the “industrial” use of oil in this country, in particular about the question whether the national oil will be exploited via a pipeline toward the Indian Ocean (and Asia) or toward the Atlantic (and Europe). Thus, the country is oil “rich” even though currently these riches primarily pay for a large-scale military conflict between Nuer and Dinka militias in the transnational fight for these resources. Finally, Southern Sudan has had “democratic” elections. The most recent independent state in Africa has made an attempt to democratically reconcile a Nuer-dominated parliament with a Dinka president, although this constellation is also considered to be one of the factors in the current unrest. In a word, the mere fact that someone is part of the Nuer or Dinka ethnic group would not automatically make that person part of a non-WEIRD sample since all WEIRD features are present here. And this seems to be true more generally across Africa and the so-called Global South where “local” people are regularly integrated into transnational and translocal connections (Ferguson, 2006, p. 106). It would be misleading to assume that any individual who happens to have a passport from Dubai, or who may be a resident of Singapore or who speaks a mother tongue other than English would automatically form part of a non-WEIRD sample. The five parameters enshrined in non-“WEIRD” are not necessarily easily operationalizable, and despite the catchy phrase we do not have good evidence to show that, for instance, being rich or being part of an industrialized economy or democratic political system would in itself affect responses in cognitive experiments. With regard to the Müller-Lyer “illusion,” for instance, the relevant parameter seems to be that of living in a “carpentered” environment (Henrich et al., 2010, p. 65) which is only indirectly connected to features such as “rich” or “industrial” and should not to be mistaken for these. Thus, the argument made by Henrich et al. (2010) provides a rough indicator to identify likely biases in existing samples but this can only be the beginning of identifying the cultural constitution of cognition, including causal cognition.

In the light of these problems I suggest an alternative: including “non-WEIRD” people in an experimental sample is not the only way of broadening the perspective and making research less culturally biased. In the light of the problem outlined above, it may in fact not be the best option. Alternatively, we should consider “harvesting” existing ethnographies in a more systematic way. By “more systematical way” I mean reconsidering the parameters for comparison. Up to now, there has been an emphasis in the literature on contrasting “the West” with “religious” and with “magical” worldviews that came to stand for “the rest” of the world. By contrast, I will be shifting our attention to the ascription of agency and the modes of time conceptualization when searching for relevant parameters. There have been few attempts to conduct “ethnographic harvesting” in a comprehensive review way (see Lillard, 1998 for an exception) since the dominant disciplinary strategy of anthropology is the in-depth single-case ethnographic monograph. My goal is to steer a middle path here, to go beyond the single-case and, instead, to explore a limited number of accessible case studies that can provide novel insights for current research which may lead the way to a broader review. There is something to be said to re-read and reconsider the earlier ethnographic literature and to discuss the relevant parameters. Standard definitions of causality refer to the relation between (a) an antecedent and a resultant item or event whereby (b) the first item has some power that necessitates the occurrence of the second. It is a reasonable hypothesis that the defining temporal dimension of sequence (a) is affected by the cultural conceptualization of time as a relevant parameter and that the defining element of (b), “necessitating with power,” is affected by cultural concepts of agency. The purpose of this enquiry into agency and time, therefore, is to facilitate research into the cultural constitution of causal cognition without making premature decisions and commitments as to what exactly constitutes relevant cultural difference with regard to any particular question. A related goal is the attempt to bring the existing ethnographic literature to bear on questions of cognition in a way that methodologically complements experimental research and that helps to theoretically “harvest” these ethnographic sources, facilitating the interdisciplinary exchange between anthropology and other disciplines investigating cognition.

## CONCEPTS OF AGENCY IN THE ETHNOGRAPHY OF CAUSALITY

The first ethnographic accounts of African people like the Zande, the Tiv, the Nuer and Dinka were compiled in the first half of the 20th century (Evans-Pritchard, 1937, 1940; Lienhardt, 1961; Bohannan and Bohannan, 1969), arguably at a point in time when members of these communities were less Westernized, less school-educated, less industrialized, less rich in consumer products and less integrated into democratic nation states – while still being far from isolated or “uninfluenced.” These ethnographic descriptions have earned fame in that they constitute the first serious attempts to specify what is different and what is similar when comparing the causal cognition of the European observer with that of the ordinary members of these groups concerned. The most widely discussed example is that of Evans-Pritchard’s (1937) work among the Azande (Zande, pl.) where he was able to distil aspects of their causal thinking by witnessing (and eliciting) their reactions to everyday and extraordinary events such as the attribution of causal agency in mishaps. The main differences that he recorded concern not the “internal” construal of causality but rather how far causal explanations are expanded and what entities are included as causal agents.

Zande grain storage baskets are mounted on poles and also serve as shady resting places for people to sit under. Occasionally the baskets collapse and hurt or even kill people who are seated underneath. Evans-Pritchard (1937) shows that Azande are well aware of the role of termites in making the poles brittle and in contributing to the collapse of grain storage baskets. However, when people get hurt in the process, the activity of the termites are not considered sufficient but the causal explanation is expanded to include the possible effect of the socially malevolent agency of witches (Evans-Pritchard, 1937, p. 69). Thereby Azande seek to explain why the storage collapsed at exactly that point in time when particular people were seated underneath. The causal cognition is expressed by Azande in a metaphor of “two spears” whereby “natural” causes and “witchcraft” can supplement one another like two spears hitting an elephant are considered equally causally affective (Evans-Pritchard, 1937, p. 74). Considering two causes with equal impact is neither weird to “Westeners” today nor was it to Evans-Pritchard back then. Consequently, Evans-Pritchard’s depiction in large parts underlines that Azande follow the same logical thought as anyone else, but that they differ with regard to the premises that they accept. The inclusion of witches as agents is such a premise and, although Evans-Pritchard would maintain that not all premises are equally valid, the extension of agency to witchcraft seems to be a matter of degree rather than kind. After all, humans universally attribute personhood (and as a consequence, causal agency) to fictitious entities. Legal persons, such as corporations, companies, and institutions (such as “the crown” or “the state”) can take political, economic, and legal action by taking political decisions, by owning property, or by taking someone else to court. The emergence of such “fictitious” corporate agents in Europe is itself intertwined with religious thinking in particular with Christian thought on the Lord’s two manifestations as Father and Son (see Kantorowicz, 1957) but the notion that non-human, religious beings are generally endowed with personhood and agency is more widely spread. Moreover, what has become classified as “religious” thought in turn follows a more fundamental “fiction” of imagining corporate social agents (the kin group, the generational set etc.). In this respect it may not be useful at all to distinguish religious from non-religious thought since this overdraws a distinction that relies on fundamentally similar processes (Bloch, 2013). The ethnographic evidence supports the view that a strict separation into two domains and two modes of thought, one religious and one non-religious, is not born out. Consider the striking similarities from the Zande and Dinka cases in which there is a seamless merger, or more precisely a refusal to draw a clear boundary, between such domains of thought.

“Thus, when a man cuts his foot either they [the Azande] do nothing or wash it and bind it with leaves, and it is only when it begins to fester that they commence to trouble about witchcraft. […] In minor ailments or at the early symptoms of an illness from which a man may be expected to recover without difficulty they think less of witchcraft and more of the disease itself and of curing it by the use of drugs.” (Evans-Pritchard, 1937, p. 509)

“A Dinka may complain of a cold or a headache without reference to Powers as the grounds of these minor discomforts. Should the cold turn to high fever, or the headache become persistent and agonizing, his thoughts will turn to the possible activity of Powers.” (Lienhardt, 1961, p. 147)

In both cases there is a seamless movement from human to suprahuman agents. In fact, both Evans-Pritchard and Lienhardt report on considerable internal cultural diversity and debate amongst Azande and Dinka who would discuss whether and when there is the need (or the justification) to refer to witchcraft. Evans-Pritchard reports that a potter whose clay pot cracks during the process of burning may attribute this to witchcraft whereas others may rather consider this a case of negligence on the side of the potter who failed to free the clay from stones that may cause cracks to occur in the process of pot-making (Evans-Pritchard, 1937, p. 77). Lienhardt’s (1961) reference to “Powers” in the above quote underlines the point. He refers to the Dinka term “*jok*” which refers to a wide class of ultrahuman agency. It may be used as a noun in singular or in plural form (then referring to distinct individual existencies of these types) but also as a more general qualifier, an attribute. So, for instance, Dinka called some forms of European technology “*turuk ee jok*” (“the European is ultra-human Power”). But they were not blurring the human vs. non-human distinction (they would not say “*turuk aa jaak,*” the Europeans are ultra-human Powers) but highlighted that agency and humanity may overlap rather than coextend (Lienhardt, 1961, p. 31). The subcategories of the general class *jok* also provide considerable flexibility. Within the category *jok* the most important powers are called *yeeth* (which has a singular form), which in the older literature would have been translated as “spirits,” and these are of two types, firstly those associated with the descent group, “clan-divinities” is the term that Lienhardt (1961) uses, and secondly those that he translates as “free-divinities” that is those *yeeth* that have proper names and who are associated with individuals rather than groups. There are not only semantic but also pragmatic aspects to these terms so that speakers may shift from implying that something “is” a Power (when giving proper names to the *yeeth*) to something “being an indication of” a Power and “a sign of ultra-human activitiy” when explaining the unexpected behavior of an animal, for instance (Lienhardt, 1961, p. 31–32). Lienhardt’s (1961) decision to translate this as “Power,” rather than limiting it to religious agents such as “spirits” reflects the fact that Dinka shift seamlessly between what Europeans may consider two completely separate domains, namely the religious and the profane. For instance when confronted with a new European gadget for the first time, they may see it as an instance of “Powers” (see above) but may drop it later when realizing that it is “merely mechnical” (Lienhardt, 1961, p. 31). Conversely, something that initially was not considered to be subject to ultra-human power but discussed as a physical and social reality (for instance ordinary rain or a slight illness) may be re-classified pragmatically as *jok* once they become out of ordinary proportion (Lienhardt, 1961, p. 147).

## “HARVESTING” THE ETHNOGRAPHY

Ethnographic evidence like that contained in Lienhardt’s (1961) monograph has for a long time been interpreted by Western scientists as a comprehensible but ultimately unwarranted and naïve extension of agency into the “invisible world.” However, some caution is in order here. For one, dealing with causality involves the universal problem that causation is not subject to direct observation but has to be inferred. The discussion as to what is considered relevant in this inference has not ceased with modern science. Moreover, the selection from a host of factors that are relevant has not begun with modern science either. Lienhardt’s (1961) account shows that this is not a case of non-Westeners readily accepting the agency of spiritual beings when something unforeseen happens. The Dinka default assumption is that there are Powers at work that enable or disable human agency in the first place. But among these powers some are better known – also with regard to their effects – than others and some remain anonymous and vague to the extent that we may no longer consider them as identifiable agents, at all, but rather as the efficacy associated with a certain place or a constellation (Lienhardt, 1961, p. 32). Some Powers are specifically labeled “*nhialic*” which links them to the domain of creation and what the European ethnographers would call ritual and religion but this does not imply that the Dinka consider them “supernatural” (because ultra-human Powers are considered to be part of the “natural” world and there appears to be no culture/nature divide as in European science). Consequently, the term is translated by Lienhardt (1961, p. 30) not as “God” or “Spirits” but as “divinity” in an attempt to catch and convey a spectrum of Powers “according to context” that are either “more substantive or qualitative, more personal or general in connotation.” Neither of the Dinka terms (*jok*, *yeeth* or *nhialic*) easily map on the English distinction between “a Power” as an agent on the one hand and a powerful situation where one event leads to the other without the interference of an agent on the other hand. Lienhardt’s (1961) problem as an ethnographer was that the two terminologies and conceptualizations inadequately map onto one another. Anthropologically speaking, however, he is struggling with a more general problem of how to understand “action” in the first place. Lienhardt (1961, p. 151) writes:

“If the word ‘passions,’ *passiones*, were still normally current as the opposite of ‘actions,’ it would be possible to say that the Dinka Powers were the images of human *passiones* seen as the active sources of those *passiones*. The practice of divination illustrates the way in which a division in experience […] is regarded as a necessary preliminary to human action. A diviner is a man in whom the division is permanently present, a Power, or Powers, are always latent within him, but he has the ability to […] letting them manifest themselves in him.”

In other words, in the Dinka way of talking about causation in terms of humans being “the object acted upon” (Lienhardt, 1961, p. 150) is a recognition that humans are always first and foremost patients. They would, for instance not talk of a man catching an illness but “the disease, or Power, always ‘seizes the man’ ” (Lienhardt, 1961, p. 150) which is why, to Lienhardt, “passiones” seems an attractive way to put it. This applies not only to divine Powers but also to places. For instance Khartoum, when being remembered as a place, is regarded as the agent – and not the mind of a human who remembers Khartoum (Lienhardt, 1961, p. 150). Similarly, there is a notion of “guilty indebtedness” whereby debt is not primarily attributed to the debtor but the creditor (or the Power directed by him), i.e., it is an activity of the giver, who is considered the guilt creator (Lienhardt, 1961, p. 150).

Dinka do not treat the inner world, the psyche, the mind or memories as interior agents that then get “extended” out but rather as the exterior acting upon them (Lienhardt, 1961, p. 149–154). Note that in this they are not altogether different from the ways in which agency and causality are considered in post-Enlightenment Europe. Dinka do not consider spirits as being materialized in Ghosts (as in the European tradition) that can be encountered in the external, material world and are independent of human experience. Modern science largely excludes conscious experience of causality altogether if there is no material correlate. However, in both cases this results in a tendency to underrate human action. This is the case for science (see below) and for the Dinka for whom “people do not choose their divinities, they are chosen by them.” (Lienhardt, 1961, p. 151). When being possessed it is “not the man but the Power” which is acting (Lienhardt, 1961, p. 148) and it is the role of a diviner to “discover a reason for the action of the Power” which in turn is related to actions of the patient (sins, omissions, and commissions) which are half-forgotten by the patient him- or herself (Lienhardt, 1961, p. 152). In other words, recognizing as to who the agent is, and for what reason, is not a trivial thing, at all, rather it requires specialist attention since there are many latent elements in one’s experiences and to discover which one is an indication of an agentive Power is by no means easy, and ordinary people may actually disagree on the diagnosis. They may murmur the word *nhialic* as an appropriate account for why something “accidental” has happened, and as if Divinity was predictable to some degree, but at the same time it is also fundamentally a recognition of the unpredictability in human life (Lienhardt, 1961, p. 54). Given this general unpredictability we may therefore conclude that ways in which Dinka pragmatically deal with this situation has many parallels with what “ordinary” Westerners do. This observation has led Lillard (1998, p. 3) not only to replace the broad notion of “Westerner” with the narrower “European American” (EA) but also to distinguish EA from the European American Social Science Model (EASSM). While EA, ordinary Westerners, may accept the influence of “nonmaterial source like spirits” on the mind, EASSM does not (Lillard, 1998, p. 3). The move, since then, from “mind” to “brain” may make it necessary to make a further distinction between the EASSM and something like an European American Neuro-Science Model (EANSM). The diversity within all cultural groups, including the Dinka, also holds for “the West.” The strategies of “ordinary Westerners” may be equidistant from “the scientific view” as non-Europeans are. Science is less than ever before a monolithic block.

The assumption that ordinary Europeans share “non-scientific thinking” with indigenous people abroad is not new. Frazer (1993/1922, p. 40) juxtaposed the Batak of Sumatra, the Baganda from Africa and midwifes from Berlin and Bavaria, as well as “malignant savages in Australia, Africa, and Scotland” (p. 13). In the meantime there are good studies on so-called “modern heathens” and others, usually considered “subcultures” of the West (Rozin and Nemeroff, 1990; Luhrmann, 1991, 2012; Medin and Bang, 2014) that substantiate this point. However, there is something peculiar here in that Westerners may in practice follow strategies not dissimilar to those described for “magic” in non-European settings but that in cross-cultural comparison “science” is claimed to be part of European culture, in fact the prototypical image of what Western culture and the concept of “culture” stands for (Wagner, 1981, p. 24). To begin with, the opposition “science-religion” is a peculiarity of a particular European history. In other words, both “science” and “religion” as separate but mutually constitutive categories are not necessarily to be found at other times and in other places (Bloch, 2013, p. 32). Therefore, “Western culture” is not to be confused with “science” but it is defined by a link between the two within a single social system that is not universal. Moreover, that which is typically considered “magical” practice need not fall on the same side of the equation as “religion” but rather on the side of science. Several early comparisons consider magic to be a precursor to science, and science to “revert” to a magical preoccupation with “inflexible regularity in the order of natural events” after a historical interlude of religious metaphysics (Frazer, 1993/1922, p. 712). In a similar vein recent contributions on causality, too, consider the “scientific” preoccupation with linear, uninterrupted causal chains a manifestation of human attempts to install an illusionary control over unpredictability and to rid oneself of unresolved questions, ultimately an expression of fear and compulsion (see Fuchs, 2008, p. 300). This makes some approaches in science look similar to magical thinking elsewhere but dissimilar to both religious metaphysical position and to minority positions in Western philosophy. In this sense we may find a similar spectrum ranging from “obsession with control” to “accepting uncertainty” both within the cultural worldview of the Dinka as well a within “the West.”

While it is useful to distinguish Euroamerican folk psychology from Euroamerican Science Models (Lillard, 1998) it is also important to note that science does not take place in a culturally neutral sphere but that it is “infected” by non-scientific ideas, for instance with regard to the attribution of agency. For the case of modern science, Deacon (2012) has recently shown that most accounts based on genetics and Darwinian evolutionism in fact show considerable continuity to pre-scientific world views in that they recreate a “homunculus” when giving causal accounts. Depending on the orientation of the researchers it is either the DNA or physiological correlates of “the brain” that are attributed with agentive power or the exterior supra-individual process of natural selection (Deacon, 2012, p. 52). His own view is an inversion of the ultimately Cartesian dualism according to which non-material aspects of the action-process need to be treated as being “on top” of, and supplementary to, physiological ones. He suggests that the “unfinished” and immaterial aspect of human action in terms of plans, aspirations/anticipations and apprehensions is best conceived of as an effective “absence” (Deacon, 2012, p. 23).

Deacon (2012, p. 14) diagnoses a deeply entrenched bifurcation in the current “Western” perspective in which the materialist Darwinian worldview that excludes the phenomenological reality of human subjectivity in causal cognition brings about “fundamentalist” tendencies that cater for this reality but at the price of largely negating or encapsulating the results of modern science. In the Dinka case we do not find this bifurcation but we also find variation within the Dinka as a cultural group as to how they interpret situations in which unusual things happen. People disagree as to whether something should be interpreted as a sign of intervention by divinity or not (Lienhardt, 1961, p. 48). The early ethnography suggests that a plurality of views is not a recent invention of the European enlightenment. It is not that THEY are governed by a religious world view while WE agonize about uncertainties. Rather, we AS HUMANS are faced with the “uncertainties and chances of [all] human life” (Lienhardt, 1961, p. 54). Lienhardt (1961, p. 54) concludes that what he observed among the Dinka was not “a pious aspiration toward resignation to the will of an ultimately benevolent personal God” but “a recognition of real ambiguities in experience.” However, that plurality need not take the form of a choice between alternative views but rather manifests itself as variations within a broader spectrum. There are again striking parallels in other African ethnographies. Evans-Pritchard, using the notion of magic that Lienhardt (1961) tries to avoid, draws a similar picture for the Azande:

“Magic may give a greater measure of success to an undertaking than would have been obtained without its use. […] Natural conditions and human knowledge of them, and skill in exploiting them, ensure a harvest of termites. The use of a magical technique is secondary […]. It cannot normally replace it. It is an aid rather than a substitute.” (Evans-Pritchard, 1937, p. 467)

There is further evidence from other parts of Africa that indicates that the “bifurcated” and “dualistic” solution of current mainstream Western science is “weird” by comparative standards. Bohannan and Bohannan (1969, p. 36) write about Tiv of West Africa, a long way away from the Dinka and Azande: “if things are going wrong, and you are not begetting enough children or getting enough money or good enough crops – if things in general are not going as well as they should – then you ‘plant’ (*tim*) an *itymbe mku*” – a sacrifice to the ancestors (see East, 1965, p. 211–214 for a detailed description). When being prompted the Tiv responded to the ethnographers that the dead parents could not actually do (read: cause) anything for you, since they were dead (Bohannan and Bohannan, 1969, p. 38) but that all other things being equal the *mku* ritual “is for good luck – to make things go more smoothly. It isn’t something you have to have to get along – but if you do not have it, there is a greater tendency for wives to leave, children to sicken and die, your luck in hunting to be poor and your crops not to amount to much. After it [the *mku*] is set up, none of these things happens to you unless something else […] intervenes. However, any *akombo* [ultrahuman agent] […] can still seize you. The *mku* can’t stop that. It is just that if nothing else happens to you, things will go well” (Bohannan and Bohannan, 1969, p. 39).

These passages show a number of things: they reflect the culturally specific ideas and practices of dealing with the universal occurrence of mishappenings, malevolence, and the vulnerability of human life. At the most general level we may say that they are a recognition and a measure with regard to the fact that the world is not an ideal place for humans to live in. There are elements of probabilistic thinking as Tiv charms set in to decrease “the tendency” for mishaps to occur and for things to go wrong. There are also elements of interventionist thinking – the assumption of natural things such as procreation and growth to happen unless there is intervention which in turn leads to the assumption that there must have been malevolent intervention when these natural processes are stopped. And there are many indications for a recognition of complex causality since measures such as putting up ancestral shrines are not seen as “definite” solutions but rather as supporting causes that some may want to do repeatedly in order to increase the effect (Bohannan and Bohannan, 1969, p. 41–43).

A common bias among WEIRD people is to insist that there are two fundamentally opposed alternative modes of access to the environment, one religious (or metaphysical) and the other non-religious (pure physical/empirical/material). However, as indicated earlier, the ethnography does not support such a separation but rather one between “confidence in the normal” and “cooption of special measures” which does not coincide with the “metaphysical/material” divide. “Confidence” may cover both metaphysical and material knowledge while “cooption,” again, can involve restricted specialized knowledge in a spectrum that covers both the metaphysical and the material. It would be less biased and more appropriate to recognize that the dominant natural science view is one such specialization which follows a logic of increasingly reducing the range of “why questions” that can be asked. The ethnographic context suggests a seamless spectrum of “why questions,” ranging from the personal and the everyday (such as: “why did I catch this disease or fail that exam?”) to the existential and universal (such as: “why do we live and why do we die?”). Moreover, religious practices and ideas are not necessarily directed toward control but also at what may be called “coping” with causes, causative agents and complex causal chains. Practices may be geared toward a better recognition (or relevation) of causal agents and causal chains, not necessarily in order to interfere with them but rather as to adapt to them and to position oneself in a way that avoids harmful effects. The practice and attitude of much of modernist science is in many ways the striking opposite. Questions that appear to be non-verifiable are deleted from the positivist scientific discourse, the question of creation (or ultimate cause) being a case in point. As Schnepf (2006, p. 90) has shown, any “why question” that is hypothetical and that involves absences tends to be excluded from the start in favor of those “why questions” that create observable facts. Questions of causality are narrowed down to questions of verification and prediction to the extent that causality is narrowly understood purely in terms of prediction and not of explanation, an account as to how something came about. “Why *p*” is made to equal the predictive “Why was it to be expected that *p*?” while excluding “How did it happen that *p*?” which would target the actual emergence and mechanism of a phenomenon in any particular case (Schnepf, 2006, p. 92–93). Note that this narrowing down of causality to its nomological and statistical sense generates resistance within “Western culture” including explicitly non-religious philosophy exemplified by Schnepf’s (2006) critique, hence generating at least as much cultural diversity as African ethnographers have noted for their cases.

What Evans-Pritchard (1940), Lienhardt (1961), and Bohannan and Bohannan (1969) were struggling to express in their ethnographic monographs indicates that we are not dealing with a shift of gear between “profane” causal thinking and a religious worldview. Rather they describe a larger underlying pattern that encompasses both. It is a notion of mediation which “enables humans to act” or of “*faire faire*” as Latour (1993, 2010) would call it (see below). One of the main problems for today’s readers of these ethnographies is that they tend to see and interpret possible “other” ways of doing things and of conceiving causality and agency in terms of those ways from which Western science has tried to emancipate itself. The ethnographic statements are primarily categorized as “religious” (or “magic”), which in a secularized world is identified with religious views in opposition to which science has emerged and against which science is gaining its own profile. We may see this as Durkheim (1912). throwing his long shadow. Durkheim (1912), it may be recalled, argued that humans can create things larger than themselves but that in the early evolutionary phase of religious life they mistook the true powers of their own society to be exterior religious powers. And since Durkheim’s times, one could argue, the so-called life sciences claim that Durkheim and the social sciences in turn mistook the powers of the collective for the powers of the genetic program of the DNA and the neural system of the brain. What shifted in these debates about agency is the place in the chain where “true agency” is identified. Creationists and geneticists usually differ on where to allocate agency and the “true cause” of things. Where they do not differ is in their conceptualization of agency as a total power, undetermined by anything prior. “Just plain folks” may locate that center of agency at the level of the individual, creationists at the level of some higher being in the sky, and many natural scientists at the level of the DNA. What they all share is that the agent is determining the patient in a mechanistic way. When represented graphically as “A–>B,” there may be considerable disagreement as to who or what needs to be put at the position of A (and B) but there is a broad cultural consensus that links positivist scientists and creationists, namely that they agree on the arrow in the middle. Lillard (1998, p. 10), following D’Andrade (1995), reproduces this consensus by conventionally glossing every arrow between items in the graphic representation of the European folk model of the mind as a “direct cause,” with arrows against the linear temporal flow labeled “influences” (not “causes”).

When we come across other ways of doing things in the African ethnography, we mistakingly align them with positions in these current debates so that they are supposed to side with religious positions in a very peculiar secularized society. However, the ethnography that I have presented above suggests something else. It suggests something that may be graphically depicted as “–>A–>” whereby A could be anything, ranging from Gods to humans and further to non-humans but whatever the As (or the Bs for that matter), their actions are enabled by being bound into a larger network and they are enabled by being conditioned by attachment as hinted at by Latour. This also applies to the actions of most ultrahuman agents who are themselves conceived of as bound and not as completely free. They do not only make things but allow things to happen and humans can try to side with them and bind themselves to ultrahuman beings so that they can benefit from the things that are being made possible.

The evidence on causality contained in ethnographic monographs is also “harvested” in a different way by a (minority) position in Western thought, most recently popularized in the works of Latour (2010) but rapidly gaining more ground. Latour (2010, p. 65) refers largely to non-religious examples that he uses to make similar points about the variability of the notion of agency: for instance the action of a speaker speaking a language (or in his terms: a language allowing someone to speak, and the speaker allowing a language to be spoken), a writer writing on a notepad, a puppeteer performing with his string puppet, a cigarette smoker smoking a cigarette. All of these can be read against the grain: a smoker thinks he is the agent when he is smoking but there may be the opposite description whereby the cigarette is forcing the agent to be smoked, not completely off limits as a description if we think of a nicotine addict. But the critical point is this: although opposite, both descriptions adhere to a view of agents being determining for what happens, except they inverse the subject-object relation, the position of agent versus patient. In the one account it is the smoker, in the other it is the cigarette. The real alternative, Latour (2010, p. 58) insists, would be a “middle voice” which reminds us of Lienhardt’s “passiones” (see above) and it may also be seen as a continuation of Gibson’s (1979) notion of the “affordances” of objects . An opposition in terms of (full) determination versus (complete) freedom produces a dualistic image of the human being as someone who is internally free (at least has the representation of freedom) while being externally completely determined (by society, the markets, global finance, the genes or whatever). But when comparing African ethnographies with “modern lives,” Latour (1993) suggests, we are not moving from a pre-modern state – in which agency is limited – to a modern state – in which agency is liberated. Rather, Latour critizises the WEIRD societies to cultivate a non-modern state in which bounded agency is denied because they ignore the fact that agency is always bounded in one way or another (in terms of role models, kinship ties, evolutionary forces etc.). This in turn leads to an inability to see and compare the different forms that bind our agency and to influence them accordingly. In other words we should concentrate on what allows us to do (*faire faire*) certain acts (as Latour, 2010, p. 58; suggests). Rituals for instance are not binding people who would otherwise be free but rather they bind them in a particular way. If they did away with the rituals their agency was bound by other forms, be it for instance the play of violent powers, of majority forces or some other structured way of organizing cooperative action. The modernist separation into pure agents and pure patients creates all sorts of conceptual problems such as the search for prime or “real” causes. In the alternative model each mediator is thought of as allowing the next piece of the chain to become the beginning of a new action itself. The mediators enable the successor in the chain to generate an effect.

Latour’s philosophy shows clear parallels with the mode of thought described in the African ethnography of (e.g., the Tiv, Azande, Dinka) but it also helps to redefine the position of these ethnographic cases in a comparative study of causality. Earlier readings of these ethnographies were comparisons between “us,” the Westerners on the route to emancipation, striving to free ourselves from all attachments that bind us, becoming free agents and “them,” the ones who are (still) bound by attachments (religion, kinship etc.). In this comparison the others will always appear positively strange because they claim that they can only live through their attachments (for instance to divine beings) that allow them to do things, thereby violating the modernist ideal to do away with all attachments that infringe on our agency. The new reading of the comparative ethnography, by contrast, realizes that “we” (humans) differ with regard to the attachments that bind us in our actions but that at the same time allow us to exist and act in the first place. Moreover, there are cultural differences to the extent that some bindings are considered to be relevant for creating responsibility in action, while others are not. Some individual agents may be considered so closely bound up with one another that they are considered to be one agent (a kinship group, a nation, a company or corporation etc.). The non-Europeans appear to the Europeans as exotic only as long as we contrast their reality of being bound and limited in their agency with an abstract ideal of a free agency. In the new comparative paradigm individuals (and possibly cultural groups as clusters of individuals) differ with regard to WHAT binds them. As I want to discuss in the remainder of this contribution the conceptualization of time is an important feature in such a comparative ethnography of causal cognition.

## CONCEPTS OF TIME IN THE ETHNOGRAPHY OF CAUSALITY

When “harvesting” earlier ethnographies for evidence to do with causality, the notion of “time” emerges as a critical domain, next to that of agency and personhood which I have discussed above. Again, Evans-Pritchard is one of the most-cited ethnographers, but in this case with reference to his ethnography of the Nuer rather than the Azande. In parallel to my discussion above we can read his ethnography as representative for a larger body of literature and, again, we can see an emerging social science critique of the universalist assumptions of the so-called “hard sciences.” As already indicated, Durkheim (1912) insisted that society was the source of all categories, including the most basic categories of time and causality – and hence the assumed importance of the new disciplines of the social sciences. His contemporaries in the social sciences elsewhere, especially in North America and Germany, were making similar arguments based on the notion of culture rather than society but found themselves in the same position to the precursors of what today are the cognitive sciences (see Bloch, 2012, p. 86). They claimed that human cognition on time varies and that this variation can be considered a proof to the importance of society and culture as opposed to mental or biological nature. It is noteworthy that the first works that made these claims were themselves compilations of earlier ethnographic work rather than field studies. This is true of Durkheim’s (1912) work on Australian Aboriginal religion and Mauss’ (1906) work of the Eskimo. The latter had argued that Eskimo seasonality, which manifests itself in large sedentary aggregations during the winter and small and dispersed mobile groups in the summer was so pronounced that it would bring about not only two modes of polities (one more hierarchical and one more egalitarian) but also two different mind-sets associated with these two seasons. Winter became associated with a greater need to seek the advice of magicians for coping with uncertainties, to conduct communal ceremonies and to convey mythology, while summer was associated with secularism, pragmatic leechcraft, peace of mind, and the economic and ritual autonomy of domestic units. Mauss (1906) relied on second-hand information and his compilation has given rise to conflicting interpretations, some leaning toward a form of ecological determinism and others supporting a view that emphasizes the relative freedom and flexibility of social forms and modes of thought even in apparently “simple” human societies (see Wengrow, 2014 for a recent interpretation). Evans-Pritchard not only had the benefit of knowing the theoretical comparative work of Mauss (1906) and Durkheim (1912) but of having the opportunity for long-term field research to investigate these matters, in this case among the Nuer of Sudan (neighbors of the Dinka). Among the Nuer Evans-Pritchard also found a marked seasonal contrast between dry river villages in the savanna where the Nuer subsist mostly on cattle-keeping and the wet season in which floods force them to aggregate and retreat to the hills where they practice agriculture and focus on communal rituals and kinship ties. All of this, Evans-Pritchard argues, is reflected in the Nuer time concepts. Their “cattle clock” is determined by daily and seasonal routines of cattle-keeping. They measure their time according to the tasks that need to be done as required by the needs of the animals (conditioned by the environment and the material world at large) rather than being “controlled by an abstract calendric system” with “autonomous points of reference” Evans-Pritchard (1940, p. 103). This “ecological” and task-oriented time management made the Nuer appear to Evans-Pritchard to be less bothered with time pressure and therefore more fortunate than those living under his home time regime. Added to this “defiance” of time is that the Nuer seem to be only interested in the past as a means to establish relative distance or nearness with regard to one another in their kinship obligations of the present. All Nuer consider one another to be genealogically related – as members of clans, major and minor lineages that have branched off from another. To know the point in time when the lineages of two individuals have split is to know the relative distance between them, the further back in time the split occurred the more distant one’s kinship link. Given the importance of time in the construction of causality, it is easy to see that reports on such “diverging” time concepts lend themselves to the interpretation that the Nuer also have different concepts of causality or that they invest less in establishing causal relations, giving less importance to chronology and to before–after relationships.

It is not only the Nuer for which variation in time concepts has been described. In fact, the notion that conceptualizations of time in “cyclical,” “reversible” or other modes of departing from a linear mode of time-reckoning have been realized elsewhere seems to have attracted the cultural imagination. It was reported upon in ethnographies (Alverson, 1978, p. 170), and picked up by philosophers (Fuchs, 2011, p. 215) and the social scientists pursuing a relativist agenda that challenges sequentiality as one of the defining features of causal relations (see above). Summarizing these debates, Maurice Bloch has recently pointed out that “The Nuer” and other ethnographies deserve a more careful reading and a more complex interpretation (Bloch, 2012, p. 91–97). He underlines that the faculty to live by imagined time regimes that are not dictated by ecology or “nature,” is an important feature that marks off humans from other species (Bloch, 2012, p. 108). This capacity to “time travel” includes the examples already given, when Eskimo and Nuer are “freezing” seasons into two modes of social and cognitive organization and two modes of grappling with problems and of seeking explanations. This capacity allows us humans to make past and future events relevant for memorizing, planning and structuring our lives. It has wide-ranging implications since the “normal rules of time and space are temporally suspended” for the benefit of imagining alternative scenarios to the ones that we find ourselves in at a particular here and now (Bloch, 2012, p. 108). However, this human ability to imagine different time regimes, Bloch (2012) reminds us, is irrespective of the fact that there is a before–after linear time reckoning still in place that is instrumental for causality. In fact, it is the particular strength of ethnographic monographs based on long-term field research (in comparison to narrowly focused survey or experimental work) that they usually contain evidence of the parallel existence of these two modes of time reckoning. Evans-Pritchard’s work on the Nuer is a case in point. He not only shows how Nuer “imagined” genealogical time and how pastoralist task oriented time helps them organize their society in a way that allows them to lead complex and satisfactory lives. He also describes scenarios and rules which show that the Nuer take account of the linear time that structures for instance the calculated give and take of cattle when negotiating bridewealth (Evans-Pritchard, 1940, p. 222, see also Bloch, 2012, p. 94). Here the marrying out of a girl in one generation will cause cattle to be transferred to the wife-givers which in the next generation in turn will cause an inverse flow of cattle when the daughters of that girl are married off with cattle going the other way. Irreversible, calculable time sequences, Bloch (2012, p. 94) argues, are underlying Nuer relationships in the everyday and they are key for the communication between the Nuer and for their ethnographers for establishing enough shared common ground to explain the subtleties of those parts of the time regime that are “imagined” and not necessarily shared culturally. On the basis of this example Bloch (2012, p. 115) formulates some general guidelines for the cooperation between anthropologists and cognitive scientists which he sees as being necessarily complementary because they target different levels of cognitive processes: anthropologists focus on the cultural imagination that differs culturally while cognitive scientists such as developmental psychologists focus on the level of time and causality concepts that are acquired early in life and are universal. The full story, however, that is provided by comprehensive ethnographies, is to recognize the co-existance of apparently “cyclical” ideas about time and an underlying before-and-after time reckoning.

Bloch’s (2012) treatment of Evans-Pritchard’s ethnography echoes an earlier attempt by Gell (1992) to summarize the rich comparative material on the time concepts of non-WEIRD people. Going back to the terminology of John McTaggart and David Mellor, Gell (1992, p. 151) distinguishes A-series from B-series time concepts whereby the former refers to the culturally harnessed experience of past–present–future (yesterday–today–tomorrow) while the latter refers to the before-and-after row of typically measurable events . This shorthand summary of time types allows Gell (1992) not only to distinguish the many different philosophical approaches to time but also to classify cultural manifestations, as found in ethnographic descriptions, that either tend toward an A-series or the B-series conceptualization.

The time concepts of the Nuer can be described in terms of an intricate combination of these two time concepts. Their “cattle clock” is an example of “concrete, immanent and process-linked” time reckoning of one action or task leading to the next, at the microscopic level. By contrast their lives at the long-term macroscopic level, like in most African societies documented in the classic ethnographies, is structured by intricate social concepts of generations, age-set and other socially “constructed” units (Gell, 1992, p. 17). The construction of a lineage and generation in a sense “immunizes” these social units of “generation,” “age set” etc. from the duration of time. A generation has no fixed number of years and age-sets can cover more or less years depending on demographic factors since a sizable group of young men need to grow up to form such a ritually constructed age-set. People belong to an age-set or a lineage not due to a specific time that has lapsed but with regard to the cultural limits set by rituals and other cultural means. The relationship between socially defined “cultural epochs” and the relationship between ancestors and present-day people is not altered by the durational intervals between them. However, these cultural rules do not undermine an understanding of a (before–after type) preceding of one epoch before the other (Gell, 1992, p. 22). Having surveyed both the comparative ethnography and the spectrum of time theories Gell (1992, p. 320) concludes that B-series time is in fact more fundamental than A-series time but he recognizes that many of his colleagues arrive at the opposite conclusion.

It is interesting to note that Gell (1992, p. 320), like Bloch, advocates a methodological bridging of the gap between the measuring approaches that focus on B-series time (time-geography approaches are his example) and the anthropologists – the “cultural/cognitive approaches” in his words that focus on A-series time. However, he also gives some hints as to why this complementarity of approaches may not be so easily achieved. Since there appears to be a direct link between debates on the conceptualization of time and those on causality we may transpose his arguments from temporality to causality in the following way: Gell’s example (1992, p. 169) relates to preparations for ceremony whereby the (B-series) sequence may be “Six moons pass before the great ceremony: one moon for fishing, one for hunting, one for making gardens, one for gathering nuts, one for visiting relatives, and then the great ceremony occurs.” What if, after 6 months, the ceremony still has not been conducted? According to Gell (1992) that is a common situation in which visitors find themselves frustrated in their communication with locals, either questioning the reliability and capability of informants or assuming that there is a completely different worldview and concept of time and causality at work. It is easy to read the statement on the right time of a ceremony as a list of causes whereby any question on “Why is there (or isn’t there) a great ceremony?” may be answered with reference to fishing, hunting, gardening, gathering, and visiting as causal prerequisites that lead to a great ceremony. However, the cognitive task is not that of counting the months and of knowing the correct sequence (B-series time) but rather to establish whether enough hunting, gathering, visiting, etc., has occurred for them to be considered “past” and “done” (A-series time). This is the situation for anyone who strives for a successful harvest. One relies on B-series time sequences of appropriate circumstances (springtime, lack of frosts etc.) that need to be in place “to cause” a good harvest (Gell, 1992, p. 173). The Nuer who leave the flooded savanna to start gardening in the hills rely on their ecological calendar for that purpose, “causing” their seasonal movement. And, presumably, at a more microscopic level their “cattle clock” works in a similar way as an orientation for the daily tasks for tending cattle that are necessary causes for a herd to survive. However, as Gell (1992) – no doubt informed by his background as an ethnographer – is quick to add, these sequences of time events (or causal events for that matter) are not by themselves decisive for human action. The ecological calendar or cattle clock may be known to be sequences that are true at all times and for everyone. In order to act, however, I need to establish where we are in the sequence and in that process agents rely on A-series type time features. What tells me that it is indeed the rainy season now and indeed time to plant or to move to the hills and so forth? The A-series time of experiencing changes from yesterday to today or from dry to rainy season or from taking the cattle out to bringing them back into the corral is decisive here. It is only a sense of the current moment that enables me to act. With reference to causality we might say that the spectrum of (immediate, distant, intermediate etc.) causes are important to know but for taking action I also need to know which of these causes need attention right now, which are the ones that are unproblematic at that particular point in time, and which are the ones that can or should be influenced at any particular moment. In Gell’s (1992, p. 174) words: “It is the farmer’s belief that springtime is truly here, and that frosts will not damage the growing shoots, [i.e., his A-series time belief, TW] which causes him set to and plant his fields.”

Gell (1992) observes that the combination of the two time series often leads to confusion when conducting ethnography but we may safely generalize this as a problem of “reading” cross-cultural data. The researcher who wants to establish when a ceremony is being held may seek an A-series answer but is actually given a B-series response or vice versa. Respondents may repeat, month after month, that the months of fishing, hunting, gardening, gathering, and visiting have to pass which may frustrate the ethnographer who actually wants to know whether the time for the ceremony has come, here and now. An observer may ask Azande what caused the death of a person but will not be given a comprehensive (B-series type) of response about contributing factors such as termites eating poles of grain storages but instead will be provided with remarks about witchcraft and the need to ask a diviner about current social conflicts because these are the factors that are relevant to clarify at that particular point in time. It is not far-fetched to recognize the different interests of ethnographers and psychologists here since the interest of the former is typically to understand the complexities of a case situation whereas the latter may be more interested to get at the more stable sequences. This is the “danger” involved when cognitive scientists read extracts from ethnographic monographs. They (like ethnographers starting their fieldwork) tend to either mistake (A-series type) comments about what is relevant “here and now” as remarks of generality or, conversely, mistake (B-series type) comments about what typically occurs as remarks of what necessarily should occur in that particular instance. Experiments geared toward general statements of “what causes what” may get interpreted by the respondents in different ways and the responses may be interpreted by those conducting the experiments in inappropriate ways. At the same time, this explains an important strength of ethnographic accounts: it is not only that they produce statements (about time or causality) that probably could not have been gained in any other way of elicitation, but rather that they provide enough context to see what type of response(s) are made as activities and events unfold. Humans effortlessly switch between the different modes of thinking about time and causality but documenting these switches with regard to events and processes unfamiliar to the observer does require considerable effort. Both, Gell (1992) and Bloch (2013) strongly underline that cultural gaps can be overcome in the process. The task of ethnographically investigating how people juggle conceptualizations of causation across different social situations is ongoing, independently of the question whether respondents are considered weird or not. Bloch (2013, p. 115) insists that ethnographers and their subjects, despite diverse explicit cultural representations, sufficiently share “core knowledge that all human beings require” for getting about their everyday life – a shared B-series, if you will that allows mutual understanding. Gell (1992) sees the possibility of understanding across “cultures” not so much in a culture-free core than in the fact that we are able to recognize “artificial” cultural roles (largely of an A-series type) through the principle of reciprocity of perspectives that allows us to understand different conceptualizations because we produce them ourselves all the time. We understand the social role played by others on the basis of our own filling in roles over and again (Gell, 1992, p. 319), exploiting a shared human facility for “excentric positionality” (Plessner, 2003, p. 364).

## CONCLUSION

In this contribution I have argued that broadening the spectrum when investigating the cultural constitution of causal cognition does not necessarily mean confronting “exotic” people with experimental tasks that were so far limited to a small WEIRD sample. Revisiting a few of the existing ethnographies can provide important insights, too, in particular with regard to differences in establishing agency and in conceptualizing time. With regard to agency, a careful analysis shows that supernatural agency does not mean that there are no universal human abilities to recognize causal chains – but that the recognized items in these chains may differ considerably nevertheless. The divide between metaphysical/material that is so pronounced in Western thinking may be much less relevant for understanding cultural diversity than the spectrum between control and accommodation. In terms of time the ethnography suggests that a co-occurance of partly conflicting time modes entertained by a group, or an individual agent, is a common feature. Moreover, there are different operations at work, that of establishing agreement about series of sequences and that of establishing whether a state in the sequence has been reached or completed. However, it is important to note that these are within-culture rather than between-culture differences. What, then, are cross-cultural differences that are likely to remain? While this awaits further concerted research beyond this contribution there are some points to note: the difference between agent and event cognition, over which much ink has been spilled in Western philosophy and psychology appears to be much less fundamental cross-culturally than commonly assumed. Similarly, there seems to be much less necessity to establish whether A-series time is more basic than B-series time than long debates in this field suggest. Ethnographic accounts show that in practice agent and event may merge beyond recognition and that both modes of time reckoning are equally involved.

It remains to be seen whether revisiting ethnographic accounts can actually correct psychological problems that have been operationalized as experiments, or even render some of the existing questions obsolete. One major corrective that ethnography can provide has already become apparent: against a dualistic image of cognitive processes made up of “unconscious quasi-mechanical cognitive processes” on the one hand and “higher, conscious thoughts, social representations and ideas” on the other hand, that is so common in psychological research, ethnography opens up perspectives that integrate both and thereby reduces “weirdness” in a number of ways. While the integration of “weird” results into the investigation of the cultural constitution of causal cognition is now a widely shared aim, the notion of “weird” in this context has received a number of different meanings: initially applied to the “aberrant” responses of non-Western peoples, it has shifted to connote the narrow and culturally rather specific sample of WEIRD respondents of most of experimental science. In this conclusion I suggest that there is, in fact, a much deeper gulf to be bridged than the one between “Western” and “non-Western” samples. It is true that ethnographic accounts have for a long time focused on non-western societies whereas experiments focused on those who are culturally close to the mind-set of cognitive scientists. However, any ethnography, independent of its location, highlights the role of *conscious* agents both in causality and in the analysis of causal cognition. We may conclude that experiments and ethnography are designed to target different aspects. There is also an element of evaluation involved and one that seems not to have changed much since the days of early ethnographic writing in the 20th century. Evans-Pritchard, it has been highlighted (Winch, 1964), may have been sympathetic to his informants but he ultimately thought that they were wrong. Materialist natural science, and a cognitive science that orients itself toward it, privileges the detection of linear causal chains (Gell’s B-series) and considers these to be “ultimately” important. By contrast, ethnography, or other methods that are experience-near, insist that it is “ultimately” the beliefs of the agents about the current state of the world (e.g., the arrival of a season, see above) that causes them to do things. As a result there is what may be called a mutual suspicion of weirdness. Enlightenment science is predicated on its fight against spiritualism and transcendentalism. Only things that were materially or energetically present were considered to be legitimate agents in causal relations following a “causal closure principle” (Deacon, 2012, p. 38) according to which nothing comes from nothing and teleological phenomena could not set causal chains in motion, neither at a situation interpersonal level nor at an evolutionary interspecies level. A lot of scientific time and energy was spent to understand “mechanical causality,” for instance in chemistry or the meteorology, which as long as they were obscure were routinely applied to gods or spirits to explain disasters or whatever deviated unexpectedly from the norm. With regard to some fields of science, in particular the inanimated processes and effects at the microscopic level this strategy has been very successful, so successful that it has been extended as a default explanation for all phenomena in which spiritual or other ultrahuman agency was proposed. Almost all of the causally relevant agents in the above-mentioned ethnographies would be brushed away on this basis, either as cognitive errors or as correct inferences that are, unfortunately, based on unwarranted, pre-scientific assumptions devoid of their necessary material basis. The model of mechanical causality has become the only accepted explanation for phenomena of living and sentient beings more generally. The assumption was that even though there are considerable gaps in the material links and causal chains to allow for a full explanation of life and consciousness, it would only be a matter of time when these gaps will be closed. In the meantime, as Deacon (2012, p. 52) has recently shown in detail, the homunculi of earlier times were re-introduced in covert forms. Activity and teleology were (and still are) routinely attributed to “the brain” or “the DNA” as “unacknowledged gap-fillers” that in fact are markers for an unfinished analysis that effectively bracket out what is most in need of explanation, namely the new process dynamics that emerges with living beings and with consciousness. Although the experience of consciously influencing causal chains is the “most commonplace phenomenon of everyday waking life” (Deacon, 2012, p. 33) the detailed scientific knowledge about the physical world (including recent advances of neural activities) suggests that plans, values and consciously set purposes are illusionary because our scientific knowledge about the physical basis does not make them easier to comprehend. However, there is growing uneasiness about this state of affairs which in effect excludes human experiential reality and the most pressing questions related to it (Deacon, 2012, p. 34). In other words, the everyday experience of humans, all humans, appears to be “weird” to the dominant science model. Conversely, a dominant science model in which the highly evolved phenomena of subjectivity and consciousness have no room, is also positively “weird” and inappropriate to explain living beings (Fuchs, 2008, p. 86).

In this context the ethnography discussed in this contribution gains particular attention: when the positive science discourse denies agency to conscious actors and dissolves it to undirected processes then this is not only questioning the particular Azande or Dinka worldview that are described here but it is also the end to any account that would be able to handle worldviews of human agents without reducing them to illusions. Similarly, when the before-and-after timeline of physical processes is said to replace meaningful identifications for “the right moment” to do something, then this renders not only the Nuer concepts of time irrelevant but more generally the human ability to decouple themselves from the here and now in which they operate.

Deacon suggests that the heart of the problem is that materialist natural sciences cannot handle “ententional” phenomena, i.e., phenomena that are causally effective (intentional, purposeful, functional, adaptive etc.) but are characterized by an absence of detectable and identifiable physical substrates. The examples he gives are of very different kinds, ranging from hemoglobin and its functions to social inventions such as money that has causal outcomes but “no essential specific physical substrate” (Deacon, 2012, p. 27–29). But examples multiply at the level that ethnographies usually tap into, namely with regard to phenomena of treating disease and dealing with misfortune that I have outlined above. Here nothing less is at stake than the realization that to a materialist science that brackets out everything that cannot be reduced to a physical or energetic process we are, as human agents, all positively “weird” in the way that we experience ourselves and the world around us. In other words, ethnographies are a constant reminder of the incompleteness of the dominant natural science account because they are populated by agents who experience themselves (and one another) as interfering in causal chains when consciously deciding when “the right moment to plant” (or to move or to burn, or whatever) has come. There is an unbridged gulf between the highly sophisticated account that the natural sciences could give on the behavior of termites on the effects of their feeding patterns onto the stability of wooden structures and the questions of meaning that Azande pose when being confronted with the physical harm that particular persons suffer at particular points in time. Deacon’s example (2012, p. 18) of a boy throwing a stone to make it skip over a surface of water underlines that point: while science may be able to provide a near to complete causal history that causes the stone to “jump” across water (in terms of what happens in the child’s muscles and brain etc.) it “leaves out what is arguably the most important fact,” namely the mental image of a skipping stone that the child may have constructed based on observing such an event elsewhere and by someone else (Deacon, 2012, p. 19). Ethnographies typically capture these images and draw these “causal” lines between events, e.g., the observation at an earlier time and place together with the pleasure of seeing a stone skip over water that is imagined by the agent. From the perspective of conscious agents these are the relevant constraints that have led to this particular event. From that perspective the geomorphological events that have placed the stone where it was found at the water shore become as insignificant as the feeding behavior of termites in African millet storage baskets – independently of whether they are known or not known.

At the end of the day this may be the most important use of ethnographies in the investigation of causal cognition: the demand for a scientific theory that satisfies both criteria, namely the critical discovery of homunculi to which agency gets attributed as a consequence of a lack of a better account *and* the insistence that phenomena such as purpose and intention are not bracketed out of consideration only because they are absent from the physical constitution at a specific place and time. This may also explain why there is less productive coexistence between ethnography and experimental work in the way that both Alfred Gell and Maurice Bloch recommend (see above). After all, this is not only a matter of combining two slightly different methods, the one more qualitative the other one more quantitative. Rather, we are dealing with indicators of a much more fundamental divide at the theoretical basis of contemporary science. A mechanistic understanding of science that is restricted to what is physically present will tend to render the purposeful behavior of conscious agents, as contained in ethnography, irrelevant. Conversely, a humanistic anthropology that focuses on the reports of conscious agents will continue to consider any advances in understanding the physical processes, typically based on experimental work, irrelevant. Unless there is a theoretical integration between the two spheres of life.

## Conflict of Interest Statement

The author declares that the research was conducted in the absence of any commercial or financial relationships that could be construed as a potential conflict of interest.

## Footnotes

1 http://www.humanservices.gov.au/customer/information-in-your-language/dinka

2 http://www.cyberspora.com/index.html (accessed August 29, 2014).

3 http://www.splmtoday.com/; http://www.nueronline.com/

## References

[B1] Alverson H. (1978). *Mind in the Heart of Darknesss. Value and Self-Identity Among the Tswana of Southern Africa.* New Haven, CT: Yale University Press.

[B2] Bloch M. (2012). *Anthropology and the Cognitive Challenge.* Cambridge: Cambridge University Press, 234.

[B3] Bloch M. (2013). *In and Out of Each Other’s Bodies. Theory of Mind, Evolution, Truth and the Nature of the Social*. Boulder: Paradigm.

[B4] Bohannan P. Bohannan L. (1969). *A Source Notebook on Tiv Religion. Cosmos, Soma, Psyche and Disease*. Yale: HRAFlex books.

[B5] D’Andrade R. (1995). *The Development of Cognitive Anthropology.* Cambridge: Cambridge University Press 10.1017/CBO9781139166645

[B6] Deacon T. (2012). *Incomplete Nature. How Mind Emerged From Matter.* New York: Norton.

[B7] Durkheim E. (1912). *Les Formes Elémentaires de la vie Religieuse. Le Système Totémique en Australie*. Paris: Presses Universitaires de France.

[B8] East R. (1965). *Akiga’s Story. The Tiv Trive as Seen by One of its Members*. Oxford: Oxford University Press.

[B9] Evans-Pritchard E. E. (1937). *Witchcraft, Oracles and Magic Among the Azande*. Oxford: Oxford University Press.

[B10] Evans-Pritchard E. E. (1940). *The Nuer.* Oxford: Oxford University Press.

[B11] Falge C. (2006). *The Global Nuer: Modes of Transnational Livelihoods*. Ph.D. thesis, Martin-Luther-Universität Halle-Wittenberg, Halle/Saale.

[B12] Ferguson J. (2006). *Global Shadows. Africa in the Neoliberal World Order.* Durham: Duke University Press 10.1215/9780822387640

[B13] Frazer J. (1993/1922).*The Golden Bough.* A study in magic and religion. Ware: Wordsworth.

[B14] Fuchs T. (2008). *Leib und Lebenswelt.* Kusterdingen: Elsevier.

[B15] Fuchs T. (2011). The Brain – a mediating organ. *J. Conscious. Stud.* 18 196–221.

[B16] Gell A. (1992). *The Anthropology of Time.* Oxford: Berg.

[B17] Gibson J. (1979). *The Ecological Approach to Visual Perception.* Boston, MA: Houghton Miﬄin.

[B18] Henrich J. Heine S. Norenzayan A. (2010). The weirdest people in the world? *Behav. Sci.* 33 61–135 10.1017/S0140525X0999152X 20550733

[B19] Kantorowicz E. (1957). *The King’s Two Bodies. A Study in Mediaeval Political Theology*. Princeton, NJ: Princeton University Press.

[B20] Latour B. (1993). *We have Never been Modern.* Cambridge: Harvard University Press.

[B21] Latour B. (2010). *On the Modern Cult of the Factish Gods.* Durham: Duke University Press.

[B22] Lienhardt G. (1961). *Divinity and Experience. The Religion of the Dinka.* Oxford: Clarendon Press.

[B23] Lillard A. (1998). Ethnopsychologies: cultural variations in theories of mind. *Psychol. Bull.* 123 3–32 10.1037/0033-2909.123.1.3 9461850

[B24] Luhrmann T. (1991). *Persuasions of the Witch’s Craft: Ritual Magic in Contemporary England*. Harvard: Harvard University Press.

[B25] Luhrmann T. (2012). *When God Talks Back: Understanding the American Evangelical Relationship with God*. New York: Vintage.

[B26] Mauss M. (1906). Essai sur les variations saisonnieres des societes eskimos. Ètude de morphologic sociale. *Année Sociol.* 9 39–132.

[B27] Medin D. L. Bang M. (2014). *Who’s Asking?: Native Science, Western Science, and Science Education*. Boston: MIT Press.

[B28] Plessner H. (2003). *Die Stufen des Organischen und der Mensch.* Frankfurt/Main: Suhrkamp.

[B29] Porteus S. (1937). *Primitive Intelligence and Environment.* New York: Macmillan 10.1037/11224-000

[B30] Rozin P. Nemeroff C. J. (1990). “The laws of sympathetic magic: a psychological analysis of similarity and contagion” in *Cultural Psychology: Essays Oncomparative Human Development* eds Stigler J. Herdt G. Shweder R. (Cambridge: Cambridge University Press) 205–232.

[B31] Schnepf R. (2006). *Die Frage nach der Ursache. Systematische und problemgeschichtliche Untersuchungen zum Kausalitäts- und zum Schöpfungsbegriff*. Göttingen: Vandenhoek & Ruprecht.

[B32] Wagner R. (1981). *The Invention of Culture.* Chicago, IL: University of Chicago Press.

[B33] Wengrow D. (2014). *Ritual, seasonality, and the origins of social inequality*. Lecture held upon invitation of the Competence Area IV and the a.r.t.e.s. Graduate School, University of Cologne, Cologne.

[B34] Winch P. (1964). Understanding a primitive society. *Am. Philos. Q.* 1 307–324.

